# Free and Conjugated Phenolics in Soaking Water from Chilean Common Bean Cultivars: Characterization and Antioxidant Potential

**DOI:** 10.3390/plants15192938

**Published:** 2026-09-25

**Authors:** Samantha Rhein, Maria Fernanda Arias-Santé, Alina Concepción-Alvarez, Miguel Ángel Rincón-Cervera, Lee A. Meisel, Basilio Carrasco, Raquel Bridi, Adriano Costa de Camargo

**Affiliations:** 1 Center for Studies in Processed Foods (CEAP), Talca 3460000, Chile; samantha.rhein@uoh.cl (S.R.); bcarrasco@ceap.cl (B.C.); 2 Institute of Health Sciences, O’Higgins University, Rancagua 2820000, Chile; 3 Institute of Nutrition and Food Technology, University of Chile, Santiago 7830490, Chile; ma.fernanda.arias@inta.uchile.cl (M.F.A.-S.); alina.concepcion@ug.uchile.cl (A.C.-A.); marincer@inta.uchile.cl (M.Á.R.-C.); lmeisel@uchile.cl (L.A.M.); 4 Department of Agronomy, Food Technology Division, University of Almería, 04120 Almería, Spain; 5 Departamento de Química Farmacológica y Toxicológica, Facultad de Ciencias Químicas y Farmacéuticas, Universidad de Chile, Santiago 8380494, Chile; 6 Instituto de Ciencias Aplicadas, Universidad Autónoma de Chile, Santiago 7500910, Chile

**Keywords:** *Phaseolus vulgaris* L., soaking water, phenolic compounds, antioxidant capacity

## Abstract

The phenolic composition and antioxidant capacity of soaking water from two Chilean common bean (*Phaseolus vulgaris* L.) cultivars, Magnum and Peumo, were analyzed. The soluble phenolics were extracted and partitioned into free, esterified, and etherified fractions. Phenolic acids and flavonoids were identified and quantified, and the antioxidant capacity was evaluated using 2,2′-azino-bis (3-ethylbenzothiazoline-6-sulfonic acid) (ABTS), ferric reducing antioxidant power (FRAP), 2,2-diphenyl-1-picrylhydrazyl (DPPH), and oxygen radical absorbance capacity (ORAC) assays. Among the phenolic compounds identified across the three fractions, rutin was the only compound detected in all of them. The free fraction showed the greatest phenolic diversity, dominated by catechin, chlorogenic acid, and *trans*-ferulic acid. Soaking water from Magnum beans exhibited significantly higher total phenolic content and greater antioxidant capacity than that from Peumo. These results suggest that bean soaking water, typically discarded as waste, is a source of phenolic compounds with high antioxidant potential and could be valorized as a functional food ingredient.

## 1. Introduction

*Phaseolus vulgaris* L., commonly known as the common bean, is a widely cultivated legume of significant dietary relevance, with large-scale production across Latin America and the Caribbean [1,2]. Due to its favorable amino acid profile, the common bean is a good and strategic source of protein for combating malnutrition in low-income populations [3,4]. Chile is home to a diverse array of *P. vulgaris* landraces and is a regionally important producer of beans in Latin America [5].

Beyond macronutrients, beans provide soluble fiber and minerals such as Fe, Cu, K, and Mg [6], as well as folate and other micronutrients that may contribute to cardiometabolic health [7]. Cardiovascular disease was responsible for approximately 19.8 million deaths worldwide in 2022 [8], underscoring the importance of dietary strategies for prevention. Bazzano et al. reported that legume consumption four or more times per week was associated with a 22% lower risk of coronary heart disease and an 11% lower risk of cardiovascular disease over 19 years of follow-up [7]. Moreover, clinical evidence specifically involving common beans (*Phaseolus vulgaris* L.) suggests beneficial effects on cardiovascular-related biochemical parameters, including LDL-cholesterol [9,10].

Health benefits are frequently attributed to the bean’s rich spectrum of phenolic metabolites [11]. These compounds quench reactive oxygen species (ROS), chelate and reduce metal ions, scavenge hydroperoxides, and attenuate LDL cholesterol oxidation, thereby curbing endothelial dysfunction and plaque instability [12,13]. Experimental studies also show that catechin-type tannins inhibit matrix metalloproteinase-1 and suppress vascular smooth muscle proliferation [14].

Phenolic compounds are secondary plant metabolites that occur in two forms: soluble and insoluble-bound. Soluble phenolics are present as free molecules or conjugated via ester or ether linkages (esterified and etherifed forms), and they are readily extracted with aqueous-organic solvents. Insoluble-bound phenolics, by contrast, remain attached to cell-wall polysaccharides or structural proteins and require alkaline or enzymatic treatments for release [15,16].

Processing conditions play a critical role in determining the extraction, release, transformation, and ultimately the bioaccessibility of phenolic compounds in beans. Among household processing practices, soaking is particularly relevant because it promotes grain hydration and facilitates the migration of water-soluble constituents, including oligosaccharides, phytic acid, and saponins, into the aqueous phase [17]. Soaking may also promote the release and partitioning of phenolic compounds into this same aqueous phase. Although soaking water is commonly discarded, the recovery of phenolic compounds from this stream suggests that it may represent an underexplored source of bioactive compounds and an opportunity to valorize an otherwise wasted agro-food by-product [18].

Phenolic compounds can occur in distinct fractions according to their association with the food matrix. These different modes of association affect phenolic solubility, stability, extractability, and release during processing, and can influence their bioaccessibility. Fraction-specific characterization, therefore, provides a more complete picture of phenolic composition than analysis of the free fraction alone, since it captures matrix-associated phenolics that conventional extraction methods may underestimate or miss entirely.

Although the antioxidant potential of black bean soaking water has been previously reported [19,20], the phenolic compounds mobilized during soaking have not been comprehensively characterized according to their free, esterified, and etherified fractions. This knowledge gap is particularly relevant for the Chilean bean cultivars Magnum and Peumo, previously identified by our research group as promising sources of phenolic compounds [21], but whose phenolic partitioning into soaking water remains unexplored. Therefore, this study systematically characterizes, for the first time, the free fraction and those released from the esterified and etherified forms and antioxidant potential of these phenolic fractions from soaking water from Magnum and Peumo.

These findings provide new insights into the transfer and partitioning of phenolic compounds during soaking and support the valorization of this underutilized aqueous by-product as a potential source of natural antioxidants. This approach is aligned with broader strategies for sustainable food processing and waste reduction. It also underscores the value of soaking water from the Chilean bean cultivars Magnum and Peumo, which hold significance not only for their nutritional properties but also for their agricultural, economic, and cultural relevance.

## 2. Results and Discussion

### 2.1. Phenolic Composition of Bean Soaking Water

Several studies have analyzed the phenolic composition and antioxidant capacity of fresh and cooked beans, extracts from bean seed coats, and sprouted beans, including their free, esterified, and etherified phenolic fractions [22,23,24]. In contrast, the soaking water, which is typically discarded after bean processing, has received comparatively limited attention [25,26]. Previous studies on Portuguese common bean cultivars have shown that phenolic compounds are released into the soaking water and that this loss varies considerably among cultivars and preparation methods. Flavan-3-ols accounted for 39.0% of the phenolic compounds identified in the soaking water and 25.5% in the coats of the soaked seeds, with (+)-catechin-3′-*O*-glucoside, procyanidin B1, and (+)-catechin being the predominant compounds [24,25].

In the present study, the total phenolic content (TPC) of the soaking water differed significantly among cultivars and phenolic fractions (Figure 1). The free fraction had the highest TPC in both cultivars, reaching 2527.8 mg GAE/L in Magnum, compared with 850.6 mg GAE/L in Peumo. The esterified and etherified fractions followed the same pattern, with values of 686.1 and 219.5 mg GAE/L for Magnum and 68.5 and 30.1 mg GAE/L for Peumo, respectively. The Magnum cultivar consistently exhibited higher phenolic concentrations across all fractions, with significant differences observed in the free, esterified, and etherified fractions (*p* < 0.0001, *p* < 0.0001, and *p* < 0.01, respectively).

HPLC-DAD analysis identified eleven phenolic compounds in the soaking water of both cultivars (Table 1), confirming the chemical diversity of this aqueous fraction. The free fraction contained the highest concentrations of several compounds, particularly in the Magnum cultivar, where catechin was the predominant compound (4120.16 μmol/L). Chlorogenic acid was detected exclusively in the free fraction of the Magnum cultivar (280.58 μmol/L), while *trans*-ferulic acid was also among the predominant phenolic acids in this fraction. This distinct pattern for each fraction and cultivar may reflect differences in the partitioning of phenolic compounds, since previous studies have reported substantial variations in the composition and distribution of phenolics among cultivars and fractions of *Phaseolus vulgaris* L. [25,27].

Evidence indicates that phenolic profiles in common bean are strongly influenced by genotype and are associated with seed coat pigmentation, with several genomic regions linked to both phenolic metabolites and seed coat color. Differences in seed coat characteristics and genotype may therefore contribute to the contrasting phenolic profiles observed between Magnum and Peumo. However, current evidence does not support a consistent pattern for the preferential location of chlorogenic acid in the free fraction. This finding is thus interpreted as a specific observation for each cultivar and fraction, under the evaluated conditions.

Rutin was the only compound detected in the free, esterified, and etherified fractions of both cultivars, although its distribution differed between them, which may be related to its chemical structure, containing several hydroxyl groups and a polar rutinoside moiety. These features may facilitate interactions with different components of the bean matrix, although the available literature does not establish a preferential partitioning of rutin into a specific fraction [28].

The hydroxyl groups and conjugated aromatic system of rutin contribute to its antioxidant properties by facilitating hydrogen-atom or electron transfer and stabilization of radical species [28,29]. Thus, rutin may contribute to the overall antioxidant capacity observed in the soaking water; however, its specific contribution cannot be established because its isolated antioxidant activity was not evaluated in the present study [29].

The presence of phenolic compounds in the different fractions indicates that the soaking process promotes their transfer from the bean matrix to the aqueous phase. The differences observed between the cultivars may be related to variations in their initial phenolic composition, seed microstructure, and the accessibility of phenolic compounds during hydration. These factors can influence both the extent of phenolic compound release and the subsequent concentration of phenolic compounds in the soaking water [30,31].

### 2.2. Comparative Phenolic Profile of Magnum and Peumo

The comparison between cultivars revealed a consistently higher overall phenolic content in Magnum. In the free fraction, the TPC of Magnum was approximately three times that of Peumo, and substantial differences were also observed in the esterified and etherified fractions. This higher overall phenolic concentration was accompanied by differences in the phenolic profile. Catechin concentration in the free fraction of Magnum was approximately 13-fold higher than that detected in Peumo, while chlorogenic acid was detected exclusively in the free fraction of Magnum. Sinapic acid was also detected only in Magnum, with its highest concentration (91.3%) observed in the esterified fraction.

Nevertheless, the phenolic profile of Peumo was characterized by higher concentrations of specific compounds. Kaempferol was more abundant in the free and etherified fractions of Peumo (54.79 and 65.25 μmol/L, respectively), while daidzein was detected at 25.46 μmol/L in the free fraction. Thus, although Magnum showed a higher overall phenolic content, the two cultivars differed in the distribution and relative abundance of individual phenolic constituents.

Nina et al. [32] characterized phenolic compounds in Chilean common beans, including Magnum and Peumo, and reported high levels of kaempferol-3-*O*-glucoside in raw seeds, which decreased after cooking. This finding suggests that processing may influence the distribution and transfer of phenolic compounds into the aqueous phase. In the present study, kaempferol was detected in both cultivars, supporting the influence of cultivar and processing conditions on the phenolic composition of bean-derived aqueous fractions.

Overall, the results indicate that Magnum generated a phenolic-rich soaking-water matrix, whereas Peumo showed a distinct profile characterized by higher concentrations of selected compounds such as catechin and rutin released from the esterified form, free and etherified kaempferol, as well as daidzein released from the free and esterified form. Therefore, cultivar selection may be an important factor when considering the recovery and potential valorization of phenolic compounds from bean soaking water.

### 2.3. Antioxidant Performance of Bean Soaking Water

The antioxidant capacity of the soaking water was evaluated using the ABTS, DPPH, FRAP, and ORAC assays, which assess complementary aspects of antioxidant activity, including radical-scavenging capacity ABTS—2,2′-azino-bis (3-ethylbenzothiazoline-6-sulfonic acid) and DPPH—2,2-diphenyl-1-picrylhydrazyl, ferric reducing power (FRAP), and oxygen radical absorbance capacity (ORAC). The combined use of these assays provides a broader characterization of the antioxidant potential of the soaking water.

As shown in Table 2, the free fraction exhibited the highest antioxidant activity in both cultivars, whereas the esterified and etherified fractions showed lower but measurable activities. Magnum soaking water consistently exhibited higher antioxidant performance than Peumo across all assays and fractions, a pattern consistent with the higher TPC observed in Magnum and suggestive of an association between phenolic abundance and antioxidant performance.

The Spearman correlation analysis, performed on the combined data from the Magnum and Peumo varieties, revealed strong and statistically significant associations between TPC and the antioxidant capacity assays FRAP, DPPH, and ORAC. Specifically, positive correlations were observed between TPC and FRAP (R = 0.979; *p* < 0.0001), TPC and DPPH (R = 0.912; *p* < 0.0001), TPC and ORAC (R = 0.870; *p* < 0.0001), FRAP and DPPH (R = 0.924; *p* < 0.0001), FRAP and ORAC (R = 0.879; *p* < 0.0001), and DPPH and ORAC (R = 0.910; *p* < 0.0001). These findings indicate that total phenolic content is the main contributor to the antioxidant capacity detected by the FRAP, DPPH, and ORAC assays in the Magnum and Peumo bean varieties, evidencing strong consistency between these methods in the evaluation of the antioxidant potential of this plant matrix.

The antioxidant activity observed in the soaking water is likely the result of the combined contribution of various phenolic constituents, rather than stemming from a single compound. Catechin, chlorogenic acid, rutin, *trans*-ferulic acid, kaempferol, and sinapic acid have been associated with antioxidant and other biological activities in previous studies [11,13,26,33,34,35,36,37,38,39,40,41,42]. However, since the soaking water represents a complex matrix, the contribution of each compound to the antioxidant activity observed in the present study cannot be determined independently. Instead, the antioxidant response may reflect the concentration, chemical structure, and interactions among various phenolic constituents and other compounds present in the aqueous fraction [43,44,45].

The relationship between phenolic composition and antioxidant activity is further supported by the consistently higher antioxidant performance of the Magnum soaking water, which also exhibited the highest TPC. Nevertheless, the higher concentration of specific compounds in Peumo, particularly catechin and kaempferol, indicates that antioxidant performance cannot be predicted solely from the concentration of an individual phenolic compound. Rather, the overall composition of the matrix and potential interactions among its constituents should be considered.

The distribution of phenolic compounds across the different fractions may also be relevant to understanding their potential bioavailability. Although phenolic compounds in the free fraction may be more readily available for absorption, those present in esterified and etherified forms may undergo transformation by intestinal microbial enzymes, presumably prolonging their activity in the gastrointestinal tract [44,45]. Overall, the presence of phenolic compounds across these different fractions suggests a potential functional effect of the bean soaking water.

### 2.4. Processing Considerations and Application Prospects

The present findings demonstrate that bean soaking water contains a diverse mixture of phenolic compounds and exhibits measurable antioxidant activity, particularly in the free fraction. These characteristics are relevant from a processing perspective because soaking water is commonly discarded despite containing compounds that may contribute to its functional properties [25,26]. The markedly higher phenolic content and antioxidant activity observed in Magnum further indicate that cultivar selection may influence the value of this aqueous fraction.

The potential application of bean soaking water should, however, be considered in the context of processing and storage stability. Although the present study demonstrates the presence of phenolic compounds and antioxidant activity, further investigation is required to determine how these properties are affected by processing conditions, storage time, temperature, light exposure, pH, and interactions with food matrices. Such information will be essential for determining whether the antioxidant potential observed in the present study can be retained under practical processing and storage conditions [25].

From a technological perspective, the results suggest that bean soaking water may represent a promising source of phenolic compounds with antioxidant potential and could be explored as a value-added ingredient in food systems, including functional beverages and other food preparations. Its potential valorization could contribute to the recovery of bioactive compounds from a currently discarded processing fraction and support more sustainable approaches to agri-food byproduct management.

The present results should not be interpreted as direct evidence of health-promoting effects in humans. Rather, they provide a chemical and functional basis for further investigation of bean soaking water as a phenolic-rich aqueous matrix. Future studies should address its stability, safety, sensory characteristics, bioavailability, and technological performance in real food systems before specific health or functional claims can be substantiated.

Overall, soaking water from the Chilean common bean cultivars Magnum and Peumo contained diverse phenolic compounds distributed across free, esterified, and etherified fractions and exhibited measurable antioxidant activity. Magnum showed the highest overall phenolic content and antioxidant performance, although Peumo contained higher concentrations of selected compounds such as kaempferol. Collectively, these findings support the potential valorization of bean soaking water as a phenolic-containing aqueous matrix and provide a basis for further investigation of its technological applications.

## 3. Materials and Methods

### 3.1. Plant Material and Sample Preparation

Two Chilean landrace bean cultivars were selected for study: Magnum and Peumo. Dry beans were soaked for 16h in distilled water at 4 °C using a bean-to-water ratio of 1:3 (*w*/*v*), as described in International Approved Methods of Analysis (AOAC) [46]. The soaking water was collected and stored at −20 °C before being subjected to phenolic extraction.

The whole seeds of the two bean varieties studied (Magnum and Peumo) were obtained from farmers in the Maule Region, central Chile. The seeds were harvested during the 2024–2025 summer season. Average seed moisture was 6–8%. Whole beans were kept at 20 °C in fabric bags without treatment against weevils. Reference material is stored at the Chilean Germplasm Bank, Instituto de Investigaciones Agropecuarias (INIA) Quilamapu, Chillan, as viable germinating seeds.

### 3.2. Extraction of Phenolic Compounds

Phenolic fractions were extracted following the method described by de Camargo et al. [47] with minor modifications. The soaking water was adjusted to a pH 2 with 6 M HCl and extracted five times with ethyl acetate (1:1, *v*/*v*). The pooled organic phases were evaporated to dryness to obtain the free phenolic fraction. The residual aqueous phase was stored at −20 °C for subsequent isolation of conjugated phenolics. For the esterified phenolic fraction, the aqueous phase was hydrolyzed with an equal volume of 2 M NaOH under magnetic stirring at 20 °C for 4 h. After neutralization to a pH of 2, the mixture was extracted five times with ethyl acetate (1:1, *v*/*v*) and the organic solvent was removed as described above. The etherified phenolic fraction was isolated by acid hydrolysis of the remaining aqueous phase with 6 M HCl at 95 °C for 45 min using a thermoregulated bath with stirring. After cooling, the suspension was centrifuged, and the supernatant extracted with ethyl acetate (1:1, *v*/*v*). All dried extracts were re-dissolved in 5 mL of methanol and stored at −20 °C until analysis.

### 3.3. Determination of Total Phenolic Content (TPC)

Total phenolics were quantified using the Folin–Ciocalteu assay [48]. A 150 µL aliquot of each phenolic fraction was transferred to a test tube, followed by 2.0 mL of Folin–Ciocalteu reagent. After 1 min, 400 µL of 7.5% (*w*/*v*) Na_2_CO_3_ and 450 µL of distilled water were added to bring a final volume of 3.0 mL. The mixture was vortexed and incubated in the dark for 30 min at room temperature. Absorbance was read at 765 nm against a reagent blank using a UV–Vis spectrophotometer (N4S UV–Vis Precise Perfect, Hinotek, Ningbo, China). Results were expressed as milligrams of gallic acid equivalents per liter of soaking water (mg GAE/L).

### 3.4. ABTS Radical Cation Scavenging Assay

A stock solution of the ABTS radical cation was prepared at a concentration of 7.00 mM by oxidation with potassium persulfate, 16 h prior to the assay. The stock solution was diluted to obtain a working solution with an absorbance of 0.70 ± 0.02 at 734 nm. Phenolic extracts were dissolved in methanol, and 3 μL of each sample or the Trolox standard was mixed with 300 μL of the ABTS working solution. After 6 min of incubation, absorbance was measured at 734 nm using a UV–Vis spectrophotometer (N4S UV–Vis Precise Perfect, Hinotek, Ningbo, China). Antioxidant activity was expressed as micromoles of Trolox equivalents per liter (μmol TE/L) [49].

### 3.5. Ferric Reducing Antioxidant Power (FRAP) Assay

The FRAP assay was carried out in 96-well microplates according to the protocol described by Bridi et al. [50] with slight modifications. The FRAP solution was prepared by mixing 10 parts acetate buffer (0.3 M pH 3.6), one part 10 mM TPTZ, and one part 20 mM ferric chloride. For each reaction, 270 μL of the FRAP solution was mixed with 30 μL of the diluted phenolic extract. The reaction mixtures were incubated for 30 min at 37 °C, and absorbance was measured at 594 nm using a microplate reader (Infinite 200 Pro, TECAN, Männedorf, Switzerland). Ferric reducing antioxidant potential was expressed as micromoles of Trolox equivalents per liter (μmol TE/L).

### 3.6. DPPH Radical Scavenging Activity

A solution of 0.06 mM DPPH in methanol was prepared, and samples were also dissolved in methanol [51]. An aliquot of 5 μL of each compound was combined with 195 μL of the DPPH solution in a microplate and incubated for 45 min, protected from light at 25 °C. Absorbance was then read at 515 nm in a microplate reader (Infinite 200 Pro, TECAN, Männedorf, Switzerland). DPPH radical scavenging activity was expressed as micromoles of Trolox equivalents per liter (μmol TE/L).

### 3.7. Oxygen Radical Absorbance Capacity (ORAC) Assay

The ORAC assay was conducted in black 96-well microplates following the method described by Bridi et al. [50]. Phosphate buffer (75 mM, pH 7.4) and a fluorescein working solution (70 nM in the same buffer; 150 µL per well) were freshly prepared and kept protected from light. Phenolic extracts were added in triplicate, and the plate was equilibrated for 20 min at 37 °C. The reaction was initiated by adding 25 µL of 10 mM AAPH (2,2′-azobis(2-methylpropionamidine) dihydrochloride) and mixing quickly. Fluorescence decay was monitored every minute for 60 min (λ_ex_ 480 nm; λ_em_ 510 nm) using a microplate reader (Infinite 200 Pro, TECAN, Männedorf, Switzerland) set to 37 °C. Net areas under the fluorescence curve were calculated and compared with the Trolox calibration curve. Results were expressed as micromoles of Trolox equivalents per liter of soaking water (μmol TE/L).

### 3.8. High-Performance Liquid Chromatography (HPLC)

The phenolic compounds in the different fractions were analyzed by HPLC-DAD using a Hitachi Chromaster 5000 system (Tokyo, Japan) equipped with a photodiode array detector and controlled via the Chromaster System Manager V1.2 software. Separation was performed on a Purospher STAR RP-18-endcapped column (250 × 4.6 mm, 5 µm; Merck, Darmstadt, Germany) protected by a Purospher STAR RP-18 endcapped guard column (4 × 4 mm, 5 µm), operating at a controlled temperature of 35 °C with injection volume of 10 µL per run, and using a gradient of methanol (A), acetonitrile (B) and aqueous formic acid solution at 0.1% (C).

The elution program resolved chlorogenic acid, *trans*-ferulic acid, sinapic acid and *p*-coumaric acids, together with catechin, rutin, daidzein, quercetin, apigenin, kaempferol and galangin, using the following solvent composition sequence at 0.8 mL min^−1^: initial conditions of 20% B and 80% C held for 10 min; transition to 7.5% A, 25% B, and 67.5% C from 10.1 to 40 min; followed by 15% A, 25% B, and 60% C from 40.1 to 50 min; 15% A, 45% B, and 40% C from 50.1 to 65 min; returning to 20% B and 80% C from 65.1 to 80 min.

Absorbance was monitored covering the spectral range of 210–700 nm and -the compounds were identified/quantified (290 nm) by comparing their retention times and UV–Vis spectra with those of the standards. External calibration was performed using a combined standard mixture prepared within the range of 5–250 µM, and individual calibration curves were generated for each analyte. The retention time (RT) and coefficients of determination (r^2^) obtained for the calibration curves of the identified reference standards were as follows: chlorogenic acid (RT= 5.49, r^2^ = 0.9874), *trans*-ferulic acid (RT = 15.11, r^2^ = 0.9927), sinapic acid (RT = 16.15, r^2^ = 0.9950), *p*-coumaric acid (RT = 12.88, r^2^ = 0.9974), catechin (RT = 5.95, r^2^ = 0.9462), rutin (RT = 11.43, r^2^ = 0.9959), daidzein (RT = 24.40, r^2^ = 0.9977), quercetin (RT = 28.27, r^2^ = 0.9952), apigenin (RT = 43.39, r^2^ = 0.9967), kaempferol (RT = 45.48, r^2^ = 0.9967), galangin (RT = 70.97, r^2^ = 0.9968). All measurements were conducted in triplicate, and analyte concentrations were reported as μmol per liter of soaked beans (μmol/L).

### 3.9. Statistical Analysis

Data are presented as the mean ± standard deviation (SD) of at least three technical replicates per fraction. Statistical differences were assessed using two-way ANOVA, followed by Tukey’s multiple comparison test, with significance set at *p* < 0.05.

Spearman’s rank correlation analysis was used to explore associations between phenolic composition and antioxidant capacity (ABTS, FRAP, DPPH and ORAC) in the free, esterified, and etherified fractions of the common bean cultivars Magnum and Peumo. All analyses were performed using GraphPad Prism^®^ version 10 (GraphPad Software, San Diego, CA, USA).

## 4. Conclusions

Our results indicate that the soaking water from the Magnum and Peumo bean cultivars is a promising source of phenolic compounds, particularly in the free fraction. The phenolic profile and antioxidant potential characterized here provide a preliminary assessment of this underutilized byproduct and provide a basis for further investigation into its valorization within the context of sustainable food processing strategies and human health. Further studies are needed to establish its suitability as a functional ingredient, including simulated gastrointestinal digestion, cellular and in vivo antioxidant activity, safety, stability, bioavailability, sensory acceptability, and performance in food matrices.

## Figures and Tables

**Figure 1:**
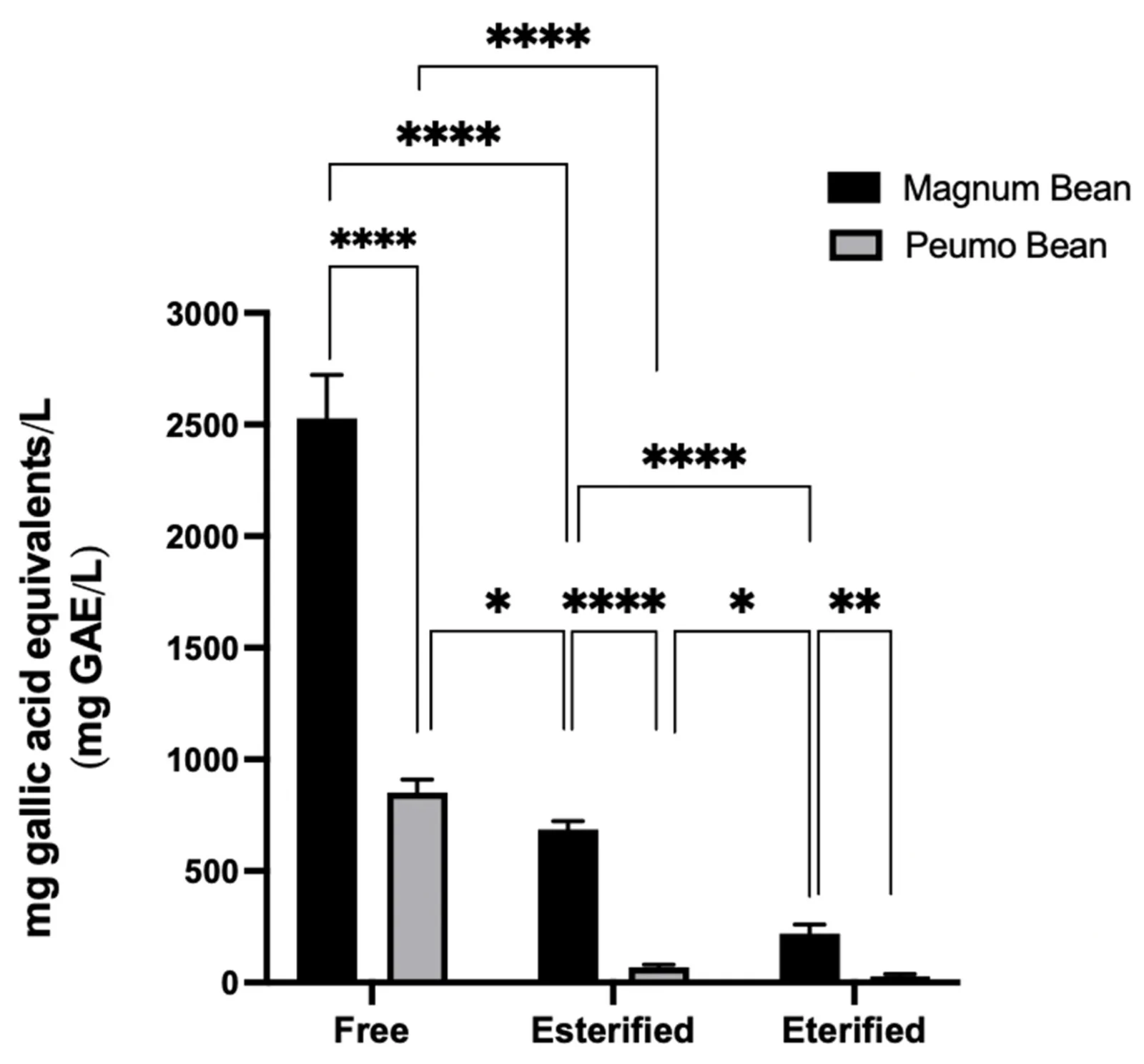
Total phenolic content in the soaking water of the common bean cultivars Magnum and Peumo. Data are shown as mean ± SD of six technical replicates for each condition (n = 6). Statistical significance was evaluated using two-way ANOVAs followed by Tukey’s multiple-comparison test (* *p* < 0.05; ** *p* < 0.01; **** *p* < 0.0001).

**Table 1:** Characterization of phenolic compounds (μmol/L) in free, esterified and etherified form present in the soaking water of Magnum and Peumo bean varieties.

	Free	Esterified	Etherified
**Chlorogenic acid**			
*Magnun*	280.58 ± 18.13	nd	nd
*Peumo*	nd	nd	nd
**Catechin**			
*Magnun*	4120.16 ± 44.38 ^A^	nd	nd
*Peumo*	294.07 ± 15.61 ^a,B^	4.13 ± 0.29 ^b^	nd
***Trans*** **-ferulic acid**			
*Magnun*	145.4 ± 2.62	nd	nd
*Peumo*	nd	nd	nd
**Apigenin**			
*Magnun*	34.25 ± 0.28	nd	nd
*Peumo*	nd	nd	nd
**Kaempferol**			
*Magnun*	38.01 ± 2.01 ^a,A^	nd	1.71 ± 0.01 ^a,B^
*Peumo*	54.79 ± 1.31 ^a,A^	nd	65.25 ± 5.81 ^a,A^
**Quercetin**			
*Magnun*	5.23 ± 0.22	nd	nd
*Peumo*	nd	nd	nd
**Rutin**			
*Magnun*	7.81 ± 0.69 ^b,B^	33.47 ± 1.55 ^a,A^	5.76 ± 0.36 ^b,A^
*Peumo*	43.31 ± 0.46 ^a,A^	23.67 ± 0.71 ^b,B^	6.23 ± 0.22 ^c,A^
***p*** **-coumarin acid**			
*Magnun*	18.58 ± 1.87 ^b^	36.27 ± 0.34 ^a,A^	nd
*Peumo*	nd	5.67 ± 0.19 ^B^	nd
**Sinapic acid**			
*Magnun*	5.47 ± 0.19 ^b^	57.24 ± 0.66 ^a^	nd
*Peumo*	nd	nd	nd
**Galangin**			
*Magnun*	23.06 ± 0.31 ^a^	nd	0.14 ± 0.06 ^b^
*Peumo*	nd	nd	nd
**Daidzein**			
*Magnun*	2.81 ± 0.30 ^a,B^	nd	nd
*Peumo*	25.46 ± 5.37 ^a,A^	2.53 ± 0.62 ^b^	nd

nd = not detected. Data are presented as mean ± SD of three technical replicates (n = 3) obtained from the soaking water of each cultivar. Statistical significance was assessed using two-way ANOVA followed by Tukey’s multiple comparisons test. Different lowercase letters in the same row indicate significant differences (*p* < 0.05) between fractions. Different uppercase letters in the same column indicate significant differences (*p* < 0.05).

**Table 2:** Antioxidant potential of soaking water of the Magnum and Peumo bean varieties.

	Free	Esterified	Etherified
ABTS radical cation scavenging activity (µmol TE/L)			
Magnum	49.74 ± 4.08 ^a,A^	7.1 ± 0.96 ^b,A^	4.6 ± 1.28 ^c,A^
Peumo	20.1 ± 11.21 ^a,B^	10.0 ± 6.3 ^b,A^	13.9 ± 3.83 ^a,A^
DPPH radical scavenging activity (µmol TE/L)			
Magnum	2152.2 ± 147.61 ^a,A^	483.18 ± 31.68 ^b,A^	209.4 ± 46.53 ^c,A^
Peumo	338.2 ± 19.78 ^a,B^	76.5 ± 6.99 ^b,B^	39.7 ± 5.89 ^b,B^
Ferric Reducing Antioxidant Power, FRAP (µmol TE/L)			
Magnum	5045.7 ± 572.2 ^a,A^	1589.7 ± 60.23 ^b,A^	649.9 ± 139.21 ^c,A^
Peumo	1796.5 ± 157.90 ^a,B^	219.8 ± 24.75 ^b,B^	71.8 ± 20.96 ^b,B^
Oxygen radical absorbance capacity, ORAC (µmol TE/L)			
Magnum	8097.7 ± 566.84 ^a,A^	1003.24 ± 148.40 ^b,B^	716.3 ± 101.13 ^c,A^
Peumo	1732.2 ± 133.08 ^a,B^	1003.2 ± 56.82 ^b,A^	363.0 ± 170.47 ^c,A^

Data are presented as mean ± SD of six technical replicates (n = 6). Statistical significance was assessed using two-way ANOVA followed by Tukey’s multiple comparisons test. Different lowercase letters in the same row indicate significant differences (*p* < 0.05) between fractions. Different uppercase letters in the same column indicate significant differences (*p* < 0.05).

## Data Availability

The data supporting the findings of this study are available from the corresponding author upon reasonable request.
